# Environmental ranking of European industrial facilities by toxicity and global warming potentials

**DOI:** 10.1038/s41598-022-25750-w

**Published:** 2023-01-31

**Authors:** Szilárd Erhart, Kornél Erhart

**Affiliations:** Joint Research Centre, European Commission, Ispra, Italy

**Keywords:** Environmental sciences, Environmental impact, Climate-change impacts

## Abstract

We present a methodology to develop the integrated toxicity and climate change risk assessment of Europe based facilities, industries and regions. There is an increasingly important need for large scale sustainability measurement solutions for company reporting with high granularity. In this paper we measure key aspects of Sustainable Development Goals in terms of human, cancer and non-cancer toxicity, ecotoxicity together with global warming impact potentials from point source pollutant releases of more than 10,000 companies and their 33,000 facilities in Europe from 2001 to 2017, by using the European Pollutant Release and Transfer Register. For our assessment, we deploy a scientific consensus model, USEtox for characterizing human and ecotoxicological impacts of chemicals and the global warming potential values from the Intergovernmental Panel on Climate Change. We discuss water and air emissions of dozens of pollutants in urban, rural, coastal and inland areas. Companies in the electricity production sector are estimated to have the largest human toxicity impact potential (46% of total) and the largest global warming impact potential (50%), while companies in the sewerage sector have the largest ecotoxicity impact potential (50%). In the overall economy, the correlation between facilities’ global warming and toxicity impact potentials is positive, however, not very strong. Therefore, we argue that carbon footprint of industrial organizations can be only used as a climate change risk indicator, but not as an overall environmental performance indicator. We confirm impact potentials of major pollutants in previous research papers (Hg accounting for 76% of the total human toxicity and Zn accounting for 68% of total ecotoxicity), although we draw the attention to the limitations of USEtox in case of metals. From 2001 to 2017 total human toxicity dropped by 28%, although the downward trend reversed in 2016. Ecotoxicity and global warming impact potentials remained unchanged in the same period. Finally, we show that the European pollutant release monitoring data quality could be further improved, as only three quarters of the toxic releases are measured in the Member States of the European Union, and a high share of toxic pollutant releases are only estimated in some countries. Of the measured or calculated toxic releases, only one third is reported according to the most robust CEN/ISO standards and about one fifth according to the least preferred other methods, like engineering judgements.

## Introduction

Earlier carbon and water footprint methodologies help assess resource consumption, carbon dioxide (CO2) emission and related climate change risks, but usually do not measure and integrate other relevant impacts, like those related to the production and use of chemicals^1^. There are well-known benefits from chemical use, still the inadequate use or excess release of chemicals may cause harmful side effects on humans and the ecosystem. Hence, in this paper we present and discuss the relevance of developing a methodology for assessing in an integrated framework the greenhouse gas emissions together with the chemical footprint of industrial facilities in terms of point source emissions.

With the current academic, regulatory and investment focus on climate change impacts, the embodied CO2 emission or carbon footprint is often used as a key performance environmental indicator for production activities^2^. To the best of our knowledge, this is the first study on greenhouse-gas (GHG) data of industrial facilities in the European Pollutant Release and Transfer Register (EPRTR) covering about 90% of point source industrial releases. We also analyze and discuss whether the GHG indicators can be used as indicators of the overall environmental performance at the industrial facility level. This may help address important policy issues, as climate change related indicators are required to report under the new European disclosure regulations from 2024, and it is important to know whether and to what extent they can be used as overall environmental performance indicators for other environmental objectives in the UN Sustainable Development Goals or EU Sustainable Finance regulatory framework (Table 1).

**Table 1:** SDGs and environmental objectives in the EU Taxonomy related to our research.

SDGs	EU Taxonomy objectives
Good health and well-being (SDG 3)	Sustainable use of water and marines (objective 3)
Clean water and sanitation (SDG6)	Transition to a circular economy (objective 4)
Sustainable cities and communities (SDG 11)	Pollution prevention and control (objective 5)
Responsible consumption and production (SDG 12)	Protection of healthy ecosystems (objective 6)
Climate action (SDG 13)	Climate change mitigation (objective 1)
Life below water (SDG 14)	

To reach our research goals we investigate the global warming footprint of facilities together with their chemical footprint. There is no consensus reached on the definition of chemical footprint, although the common understanding is that the chemical footprint should describe the (potential) impacts from hazardous chemicals from firms, facilities, industries or nations^1,3,4^. For ecological impacts, it has been suggested to build a definition on the dilution needed to avoid freshwater ecological impacts^3^. Increased chemical use is causing a growing number of environmental problems, and chemical products are pervasive in societies within animal and crop-based agriculture, in industrial processes and in households. National environmental targets, as well as the global chemical-related goals in the 2030 Agenda call for the monitoring of chemical use and emissions^5^. Investors, consumers, regulators, banks and other financial intermediaries increasingly need Environmental Social and Governance (ESG) indicators including chemical and carbon footprint information to make decisions. We present results on the company facility level and further statistics on various pollutants and major industries across EU regions.

The Protocol on Pollutant Release and Transfer Registers (PRTRs) was agreed on by international parties to register environmental footprints across regions and times. PRTRs are increasingly used as a fundamental data source for chemical footprint research^4,6,7^. However, the large and increasing number of pollutants makes it more difficult for non-expert public users to understand which substances are of greatest concern in their communities or globally. The impact of substances is best characterized when environmental release data of substances are combined with their properties in terms of global warming and toxicity impact potentials. Earlier papers found that pollutant registers may not provide critical information on the real impact of pollutant released by disclosing weight of pollutant releases, as severity of releases per emitted kilogram significantly differ by pollutants. Hence, here we provide more context with quantitative data to increase usability of these registers, as suggested by earlier authors^8^.

Using data from the Swedish Pollutant Release and Transfer Register on emissions to air and water from Swedish point sources, and characterization factors (CFs) from the USEtox model, earlier research papers suggested the aggregated impact potentials for human toxicity and ecotoxicity as key metrics to measure chemical footprints on the national, pollutant and facility level^4,9,10^. The first calculations showed that zinc contributed most to the impact potentials both for human toxicity (in the range of 30–60% of the total impact), and ecotoxicity (60%) in Sweden in 2008.

Earlier macroscopic studies on Europe also found zinc compounds and some other metals as the substances with largest contribution to toxicity in Europe in mid 2000s^1,3^. The Japanese PRTR was used for an integrated input-output analysis and structural decomposition analysis, to identify the socio-economic determinants of changes in toxicological footprint^11^. The results showed that the overall toxicity from the Japanese industrial sectors decreased by 49% from 2011 to 2015.

The aim of this study is threefold. First, it investigates the carbon footprint together with the chemical footprint of European industrial facilities. For the chemical footprint assessment, impact potentials are calculated for human toxicity and ecotoxicity associated with European point source emissions by pollutants, using the latest USEtox 2.12, a model based on scientific consensus providing midpoint characterization factors for human toxicological and freshwater ecotoxicological impacts of chemical emissions in life cycle assessment, developed under the auspices of the United Nations Environment Program (UNEP) and the Society for Environmental Toxicology and Chemistry, (SETAC) Life Cycle Initiative. For the carbon footprint calculations, we use the point-source GHG releases of industrial facilities in the EU and the IPCC (Intergovernmental Panel on Climate Change) values for global warming potential of greenhouse gases. Analyzing the carbon and chemical footprint in an integrated framework helps investigate whether and to what extent carbon footprint can be used as an overall environmental performance indicator for non-climate related environmental objectives in the UN Sustainable Development Goals or EU Sustainable Fiance regulatory framework. Second, our study attempts to increase the precision of earlier chemical footprint calculations by using population grid and distance-to-sea-coast data to better connect pollution data with the USEtox model on the sub-compartment level, following the methodology presented for Sweden^10^. Third, it assesses chemical footprint at various levels from pollutants to industries, company facilities, geographic regions and across times, which was not discussed in earlier research. Our research enhances exchangeability and acceptability of exposure knowledge within and across EU chemicals-related policies^12^.

Our research supports the achievement of global environmental policy goals by presenting a new set of environmental indicators for monitoring industrial facilities across regions and times. In particular, our indicators can be used for measuring progress on a company or its production facility level towards the United Nation’s Sustainable Development Goals (SDGs) and the environmental objectives in the EU Taxonomy regulation (2020/852 of the European Parliament and of the Council of 18 June 2020). Pollutant release data can help measure and monitor progress both towards specific of these goals and multitudes of them^13,14^. From the six environmental objectives in the EU Taxonomy regulation, at least five can be directly related to pollutant release assessment, and from the UN SDGs, six goals can be identified (Table 1). In particular, SDG Target 12.4 on environmentally sound management of chemicals, and Pollution prevention and control (EU Objective 5) are likely the most closely aligned with toxic impacts of pollutants. Climate Action (SDG 13) and Climate Change Mitigation (EU Taxonomy Objective 1) can be also assessed, as greenhouse gas releases are reported by the facilities. The EU Taxonomy Objective 3 (Sustainable Use of Water and Marines) and SDG6 (‘Ensure availability and sustainable management of water and sanitation for all’) or Goal 3 (’Ensure healthy lives and promote well-being for all at all ages’) are all influenced by pollutants released into water, air and soil, which are registered in the E-PRTR. Target 6.3 (‘By 2030, improve water quality by reducing pollution, eliminating dumping, and minimizing release of hazardous chemicals and materials, halving the proportion of untreated waste water and substantially increasing recycling and safe reuse globally’) is another specific example of relevant SDG targets. E-PRTR data can also support measure progress toward Target 3.9 ( ‘By 2030, substantially reduce the number of deaths and illnesses from hazardous chemicals and air, water, and soil pollution and contamination.’). Although the E-PRTR does not register mortality or health problems from hazardous chemicals, pollution or contamination, it can support constructing and monitoring of indicators to measure the related risk exposure by matching emission quantity, toxicity and global warming potential information on the substance level.

The European Commission’s president declared in her political manifesto that ‘Europe needs to move towards a zero-pollution ambition’ and she ‘will put forward a cross-cutting strategy to protect citizens’ health from environmental degradation and pollution, addressing air and water quality, hazardous chemicals, industrial emissions, pesticides and endocrine disrupters.‘

Our methodology helps broaden the coverage of corporations’ environmental assessment. Most common Environmental standards (ISO—International Organization for Standardization, GRI—Global Reporting Initiative, SASB—Sustainability Accounting Standards Board) fit and are available only for the largest companies. Furthermore, key Environmental Social and Governance (ESG) rating providers, Thomson Reuters, Sustainalytics, MSCI—Morgan Stanley Capital International, Bloomberg cover only few thousands of the largest corporations listed on international stock exchanges^15^. These rating institutions rely mostly on sustainability reports, annual reports, websites, and other public sources, as well as on company direct contact. In addition, the content and quality of publicly disclosed environmental and sustainability reporting varies, and reports focus mainly on carbon dioxide emission and are generally better for larger issuers^16^. Our methodology helps to broaden the coverage of ESG assessments to the major industrial polluters (about 10 thousand companies in Europe) and allows a desegregated facility and regional level thematic investigation of their sustainability risks, which is not possible when using the consolidated company level sustainability reports.

The rest of the paper is structured as follows. “Results” section presents empirical results on pollutant, industry and facility level. “Discussion” section discusses the methodological framework and “Methods” section details the limitations of our empirical set-up. “Conclusions” section concludes.

## Results

Pollutant released by industrial facilities impact on humans and the environment. The seriousness of global warming or health and ecological consequences can be underestimated if only the quantity of pollutants is used^17^. The global warming potential is strongly dependent on the chemicals’ radiative efficiency, in other words their ability to absorb energy and how long they stay in the atmosphere, known as their lifetime. The health consequences at the societal level depend also on the release media, length of exposure and population density^18^. Our results are presented here in terms of global warming impact potential, human toxicity and ecotoxicity impact potentials of point source emissions in Europe by major pollutants in the E-PRTR regulation, by industries and across regions. Impact potentials are measured in carbon dioxide equivalents (CO2eq) for the global warming potentials, in Comparative Toxic Units for human health (CTUh) and ecotoxicity (CTUe), respectively. It should be noted that CTUh and CTUe values, calculated by the USEtox model, cannot be directly compared, as they are measured on different scales and in different units.

### Global warming and toxicity impact potentials by pollutants and industries

The global warming potential is expressed in carbon dioxide equivalents (CO2eq) and allows comparisons of different gases in terms of climate change impact potential. It measures how much energy per unit weight (kg or ton) the gas can absorb over a certain period of time, compared to the unit weight of carbon dioxide. This way emissions can be added and compared across gases, sectors, regions, times or greenhouse gases. The characterization factor for human toxicity impacts (human toxicity potential) is expressed in comparative toxic units (CTUh), which estimates the increase in morbidity in the total human population. Its unit [CTUh per kg emitted] is defined as the disease cases per kg emitted. For ecotoxicity, impact potentials are expressed in comparative toxic unit for freshwater ecosystem (CTUe), which provides an estimate of the potentially affected fraction of species (PAF) integrated over time and volume per unit mass of a chemical emitted.

The global warming potential impact from industrial point source emissions is mostly related to carbon dioxide (CO2) releases of the facilities (96% of the total) in the EU Member States, in 2017, (Table 2). Methane emission is the second largest contributor with 2%. Above average share of Methane in greenhouse gases are reported in Ireland (5%), Malta (16%), Luxembourg (8%), Poland (7%), Slovenia (9%). Emissions of certain greenhouse gases (Sulphur hexafluoride (SF6), Perfluorocarbons (PFCs), Hydro-fluorocarbons (HFCs)) are reported by a smaller fraction of EU Member States.

Human toxicity impacts are dominated mostly by mercury compounds in the EU as a whole, accounting for 76% of the total impact potential in 2017 (Table 2). An important risk of further mercury emissions has been already highlighted by earlier research due to mercury’s bio-accumulation properties in organisms and humans during their lifetime^19^. Hence, mercury concentrations usually increase when moving up the food web. Our trend analysis in a later section of this study shows that mercury’s annual emission was stable in the period from 2001 to 2017.

**Table 2:** Human toxicity, ecotoxicity and global warming impact potentials by major pollutants (in % of the total CTUh, CTUe and CO2 equivalent, respectively, 2017).

Pollutant	AT (%)	BE (%)	BG (%)	CY (%)	CZ (%)	DE (%)	DK (%)	EE (%)	EL (%)	ES (%)	FI (%)	FR (%)	HR (%)	HU (%)	IE (%)
**Human toxicity impact in % of the total**															
Arsenic and compounds (as As)$^{\text {a}}$	0	1	1	1	2	0	1	7	2	1	2	1	1	8	3
Cadmium and compounds (as Cd)$^{\text {b}}$	3	2	1	2	1	0	0	3	3	3	0	4	4	0	0
Chromium and compounds (as Cr) $^{\text {b}}$	0	1	3	0	0	1	0	1	1	1	3	1	4	8	0
Lead and compounds (as Pb)$^{\text {d}}$	5	25	2	0	5	3	0	30	3	9	2	11	0	11	0
Mercury and compounds (as Hg)$^{\text {e}}$	82	58	92	83	89	92	94	43	81	67	78	57	65	51	93
Zinc and compounds (as Zn)$^{\text {f}}$	10	12	1	13	2	3	2	17	10	18	13	25	26	21	3
Largest contributions in % of total	100	100	100	100	100	100	98	100	99	99	98	99	100	99	100
**Ecotoxicity impact in % of the total**															
Arsenic and compounds (as As)$^{\text {a}}$	0	0	1	0	1	0	1	3	1	0	0	0	0	1	1
Cadmium and compounds (as Cd)$^{\text {b}}$	1	4	26	13	4	2	0	11	4	6	4	6	7	1	1
Chromium and compounds (as Cr)$^{\text {c}}$	0	4	10	3	1	3	1	6	5	4	3	2	6	7	0
Copper and compounds (as Cu)$^{\text {g}}$	3	2	4	1	3	3	2	1	3	4	3	4	2	2	5
Lead and compounds (as Pb)$^{\text {d}}$	0	0	0	0	0	0	0	1	0	0	0	0	0	0	0
Mercury and compounds (as Hg)$^{\text {e}}$	0	0	0	0	0	0	0	0	0	0	0	0	0	0	0
Nickel and compounds (as Ni)$^{\text {h}}$	10	7	12	51	16	11	12	9	27	26	15	12	3	12	9
Zinc and compounds (as Zn)$^{\text {f}}$	85	82	47	30	74	80	84	69	60	58	70	74	81	76	85
Largest contributions in % of total	100	100	100	100	100	100	100	100	100	98	95	99	100	100	100
**Climate change impact potential in % of the total**															
Carbon dioxide (CO2)	99	97	96	100	100	98	96	100	98	96	94	95	98	100	94
Hydro-fluorocarbons (HFCs)	0	0			0	1	0		0	0	0	1			0
tMethane (CH4)	1	0	4	0	0	1	4	0	1	3	1	2		0	5
Nitrous oxide (N2O)	0	2	0	0	0	0	0		1	1	5	2	2	0	0
Perfluorocarbons (PFCs)		0				0			0			0			0
Sulphur hexafluoride (SF6)						0				0		0			0

**Pollutant**	IT (%)	LT (%)	LU (%)	LV (%)	MT (%)	NL (%)	PL (%)	PT (%)	RO (%)	SE (%)	SI (%)	SK (%)	UK (%)	EU 28 (%)	
**Human toxicity impact in % of the total**															
Arsenic and compounds (as As)$^{\text {a}}$	7	0	0	1	19	4	1	2	1	2	0	0	2	2	
Cadmium and compounds (as Cd)$^{\text {b}}$	3	0	0	0	0	3	1	14	2	1	1	1	1	2	
Chromium and compounds (as Cr)$^{\text {b}}$	10	18	0	3	68	1	0	4	4	2	2	0	2	1	
Lead and compounds (as Pb)$^{\text {d}}$	7	0	0	0	0	15	6	7	16	0	9	83	17	10	
Mercury and compounds (as Hg)$^{\text {e}}$	59	0	10	93	1	61	84	63	54	57	87	10	63	76	
Zinc and compounds (as Zn)$^{\text {f}}$	14	72	90	3	10	15	5	10	17	34	1	4	14	9	
Largest contributions in % of total	99	91	100	99	98	99	96	99	95	98	100	100	99	100	
**Ecotoxicity impact in % of the total**															
Arsenic and compounds (as As)$^{\text {a}}$	1	0	0	0	1	1	0	0	0	0	0	0	0	1	
Cadmium and compounds (as Cd)$^{\text {b}}$	22	0	0	4	0	1	15	10	1	5	1	36	3	9	
Chromium and compounds (as Cr)$^{\text {c}}$	9	4	0	4	22	1	2	8	4	1	5	2	2	4	
Copper and compounds (as Cu)$^{\text {g}}$	1	2	0	6	1	2	1	2	12	2	3	0	7	4	
Lead and compounds (as Pb)$^{\text {d}}$	0	0	0	0	0	0	0	0	0	0	0	8	0	0	
Mercury and compounds (as Hg)$^{\text {e}}$	0	0	1	0	0	0	0	0	0	0	0	0	0	0	
Nickel and compounds (as Ni)$^{\text {h}}$	18	9	0	24	14	13	6	35	7	7	14	3	14	14	
Zinc and compounds (as Zn)$^{\text {f}}$	47	86	99	62	62	81	75	42	76	83	77	50	72	68	
Largest contributions in % of total	98	100	100	100	100	100	100	98	100	99	100	98	99	99	
**Climate change impact potential in % of the total**															
Carbon dioxide (CO2)	96	95	92	99	84	97	92	96	95	99	91	99	94	96	
Hydro-fluorocarbons (HFCs)	0					0	0	0		0	0	0	0	0	
tMethane (CH4)	2	1	8	1	16	1	7	3	4	1	9	1	4	2	
Nitrous oxide (N2O)	0	4		1		2	1	1	1	1	1		1	1	
Perfluorocarbons (PFCs)	1												0	0	
Sulphur hexafluoride (SF6)	0									0			0	0	

This table shows the aggregated toxicity and global warming impact potentials by pollutants. The emissions in kilograms ($E_{ijk}$) of substances (i) in the EPRTR have been multiplied by their USEtox 2.12 toxicity characterisation factors ($CF_{ijk}$), by the global warming potential value ($GWP_{i}$) and aggregated across all substances, release media (j) and release sub-media (k). GWP values by greenhouse-gases reported under EPRTR Methane (CH4): 25 co2eq, Carbon dioxide (CO2): 1 co2eq, Hydro-fluorocarbons (HFCs): 7462 co2eq, Nitrous oxide (N2O): 298 co2eq, Perfluorocarbons (PFCs): 9795 co2eq, Sulphur hexafluoride (SF6): 22800 co2eq

$$^{\hbox {a}}$$ As As(V).

$^{\hbox {b}}$ As Cd(II).

$^{\hbox {c}}$ As Cr(VI).

$^{\hbox {d}}$ As Pb(II).

$^{\hbox {e}}$ As Hg(II).

$^{\hbox {c}}$ As Pb(II).

$^{\hbox {d}}$ As Cr(VI).

$$^{\hbox {e}}$$ As As(V).

$^{\hbox {f}}$ As Zn(II).

$^{\hbox {g}}$ As Cu(II).

$^{\hbox {h}}$ As Ni(II).

The World Health Organization (WHO) includes mercury in its list of chemicals of major public health concern^20^. There is some degree of variation across Member States in terms of toxicity impacts of pollutants, but in general zinc and lead compounds are being the pollutants with considerable, 9–10% impact potential of the total on average.

The pollutants with the largest contribution to ecotoxicity was zinc in 2017 (68% of the total) together with some other metals confirming earlier results^1,3^.

On the industrial level the largest estimated human toxicity footprint was estimated for the Production of electricity (46%) followed by Manufacturing of basic iron, steel, and ferro-alloys (20%) and Manufacturing of cement (10%) in 2017 (Table 3). As for ecotoxicity, Sewerage (50%) is the industry with the largest estimated chemical footprint. Beyond having large chemical footprint, electricity production is the sector with the largest global warming potential (50% of the total). This result highlights the importance of managing the electricity production sector’s environmental footprint. It should be added, however, that electricity is a major input to other sectors’ production and therefore impact potentials are indirectly caused by the electricity demand from companies in other sectors. In the same vein, the ecotoxicity impact of the sewerage sector is dependent on activities in other industrial sectors.

**Table 3:** Human toxicity, ecotoxicity and climate change impact potentials by NACE sectors (2017, % based on CTUh, CTUe and CO2 equivalent)

Industry	AT (%)	BE (%)	BG (%)	CY (%)	CZ (%)	DE (%)	DK (%)	EE (%)	EL (%)	ES (%)	FI (%)	FR (%)	HR (%)	HU (%)	IE (%)
**Human toxicity impact in % of the total**															
Manufacture of cement	27	15	2	87	5	9	44	0	18	14	5	8	84	50	61
Manufacture of basic iron and steel and of ferro-alloys	51	30			6	12			3	22	30	38		14	
Production of electricity	0	0	91	12	69	69	33	95	53	26	1	7	0	0	21
Sewerage	4	1	4	1	1	1	3	0	1	2	1	1	3	8	6
Treatment and disposal of non-hazardous waste	0	10	0	0	0	0	17	5	0	0	0	7		7	11
Treatment and coating of metals	0	1			0	0				2		0		11	
Mining of iron ores											0				
Manufacture of pulp		2				0		0		1	2	3	0	0	
Manufacture of other inorganic basic chemicals	2	1	0			2		0	0	11	40	7		0	
Water collection, treatment and supply			0							0		0	3	0	
Largest contributions in of total	84	59	97	100	81	93	97	100	75	78	79	72	91	91	99
**Eco toxicity impact in % of the total**															
Manufacture of paper and paperboard	5	3			2	0				0	51	2		0	
Manufacture of basic iron and steel and of ferro-alloys	6	16			9	5			13	10	9	15		70	
Production of electricity	1	0	1	65	13	3	0	82	11	9	0	4	0	4	0
Sewerage	84	29	77	27	49	58	99	11	59	53	16	33	16	21	94
Treatment and disposal of non-hazardous waste	0	1	0	0	0	1	0	1	0	0	0	0		1	0
Water collection, treatment and supply			2							10		2	72	1	
Mining of other non-ferrous metal ores			7						0	0	3	0			6
Manufacture of pulp		2				0		4		2	11	3	0	0	
Manufacture of other inorganic basic chemicals	1	29	0			3		0	0	1	1	3		0	
Manufacture of refined petroleum products	1	0	0		0	1	0	1	17	3	1	5	2	0	0
Marine aquaculture															
Largest contributions in of total	97	80	86	92	73	72	99	100	100	88	91	69	89	97	100
**Global warming impact potentials in % of the total**															
Production of electricity	26	34	70	69	67	59	47	50	68	54	11	27	28	17	69
Manufacture of refined petroleum products	12	14	6		1	4	6	4	12	12	6	9	20	8	2
Manufacture of basic iron and steel and of ferro-alloys	13	11			5	7				5	3	19		5	
Manufacture of cement	11	6	3	30	5	5	21	2	13	11	2	9	31	6	18
Steam and air conditioning supply	4		13		17	6		34			26	2		5	
Treatment and disposal of non-hazardous waste	5	4	2	0	0	3	8	1	2	3	1	11		2	8
Manufacture of other organic basic chemicals	1	9			1	5				1	1	3			
Manufacture of paper and paperboard	11	2			0	1				1	21	3			
Largest contributions in of the total	83	82	94	100	96	90	82	92	94	88	71	82	79	43	97

Industry	IT (%)	LT (%)	LU (%)	LV (%)	MT (%)	NL (%)	PL (%)	PT (%)	RO (%)	SE (%)	SI (%)	SK (%)	UK (%)	EU 28 (%)	
**Human toxicity impact in % of the total**															
Manufacture of cement	3	0		93		0	10	5	1	2	69	0	6	10	
Manufacture of basic iron and steel and of ferro-alloys	46		100			40	10	5	53	28	11	92	40	20	
Production of electricity	8			0	89	19	56	32	34	1	0	0	29	46	
Sewerage	11					6	0	5	6	4	2	0	4	2	
Treatment and disposal of non-hazardous waste	2	0	0		0	11	0	0	0	0	0	0	1	1	
Treatment and coating of metals	0	28					0		0				1	0	
Mining of iron ores										19				0	
Manufacture of pulp								24		7		0	0	1	
Manufacture of other inorganic basic chemicals	3					0	0	0	0	0			8	3	
Water collection, treatment and supply	4	43		7			0	0	0		0	0	0	0	
Largest contributions in of total	79	71	100	99	89	76	77	71	94	60	82	92	90	83	
**Eco toxicity impact in % of the total**															
Manufacture of paper and paperboard	1					0	5	4		33	6	1	0	4	
Manufacture of basic iron and steel and of ferro-alloys	6		100			4	7	0	8	6	15	63	5	7	
Production of electricity	2			0	35	0	4	3	0	4	0	0	3	4	
Sewerage	59					83	23	72	54	20	60	0	59	50	
Treatment and disposal of non-hazardous waste	3	0	0		0	1	1	0	0	0	0	0	6	2	
Water collection, treatment and supply	15	97		100			1	5	3		16	1	1	5	
Mining of other non-ferrous metal ores							49	1	31	1				7	
Manufacture of pulp								6		33		3	0	2	
Manufacture of other inorganic basic chemicals	9					0	0	0	0	0			1	3	
Manufacture of refined petroleum products	1	0				1	0	4	2	0		23	1	2	
Marine aquaculture					62								9	2	
Largest contributions in % of total	95	97	100	100	97	90	91	94	100	98	98	92	86	88	
**Global warming impact potentials in % of the total**															
Production of electricity	59			52	84	44	66	51	56	0	78	14	51	50	
Manufacture of refined petroleum products	13	30				12	1	10	6	6		11	9	7	
Manufacture of basic iron and steel and of ferro-alloys	6		34			8	4		10	7		31	8	7	
Manufacture of cement	8	13		39		1	6	11	14	5	10	11	4	7	
Steam and air conditioning supply	1	5		7			6		1	28		5	0	5	
Treatment and disposal of non-hazardous waste	3	0	45		16	9	0	5	2	0	4	0	11	4	
Manufacture of other organic basic chemicals	2					7	1	0		1			2	3	
Manufacture of paper and paperboard	1					1	2	4		25	3		0	2	
Largest contributions in % of the total	93	49	79	99	100	81	85	81	89	73	95	71	85	86	

There is obvious variation of the results on the Member State level in the European Union, partly due to differences of national economic structures, in particular in case of ecotoxicity. The largest contribution to ecotoxicity is calculated for Manufacture of paper and paperboard (33%) and Manufacture of pulp (33%) in Sweden, while for Mining of other non-ferrous metal ores in Poland (49%) and Romania (31%). The largest emitter facilities are further discussed in a later section of this study.

**Figure 1:**
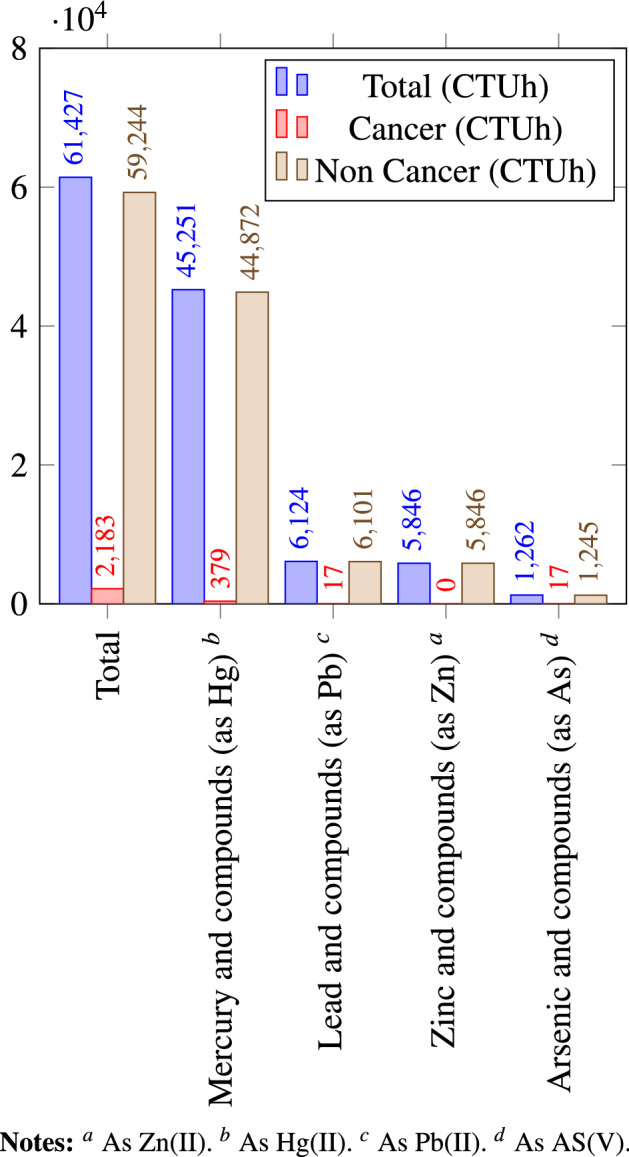
Contribution of substances to cancer, non-cancer and total human toxicity (CTUh), emitted from point sources reported under E-PRTR to air and water (2017), characterized with USEtox 2.12. Only the total contribution and the four substances with largest contributions are shown.

The USEtox model allows differentiation between cancer and non-cancer characterisation factors, assuming equal weighting between cancer and non-cancer due to a lack of more precise insights into this issue. We decomposed the results similarly to an earlier study on Sweden^10^, and found that non-cancer human toxicity dominated the aggregated human toxicity impact potentials in Europe. Non-cancer human toxicity impact potential was 59,200 CTUh of the total 61,400 CTUh in 2017 calculated with USEtox 2.12, and cancer related CTUh was only a fraction of the total, 2200 CTUh, (Fig. 1). When filtering our cancer toxicity results with the USEtox 2.12 model by pollutants and industries, we found that Chromium compounds and Polycyclic aromatic hydrocarbons (PAHs), Mercury compounds and PCDD + PCDF (dioxins + furans) have the largest cancer toxicity impact potential in Europe. The production of electricity, mining of hard coal, sewerage, manufacture of other inorganic basic chemicals are the industries with the largest cancer toxicity impact potential. Using a sample of 52 mobile phones manufactured between 2000 and 2013, it was demonstrated with the USEtox model that toxicity increased with technology innovation and chromium showed the most significant risk for both cancerous and non-cancerous diseases^21^.

### Regional mapping of European toxicity and global warming potentials

**Figure 2:**
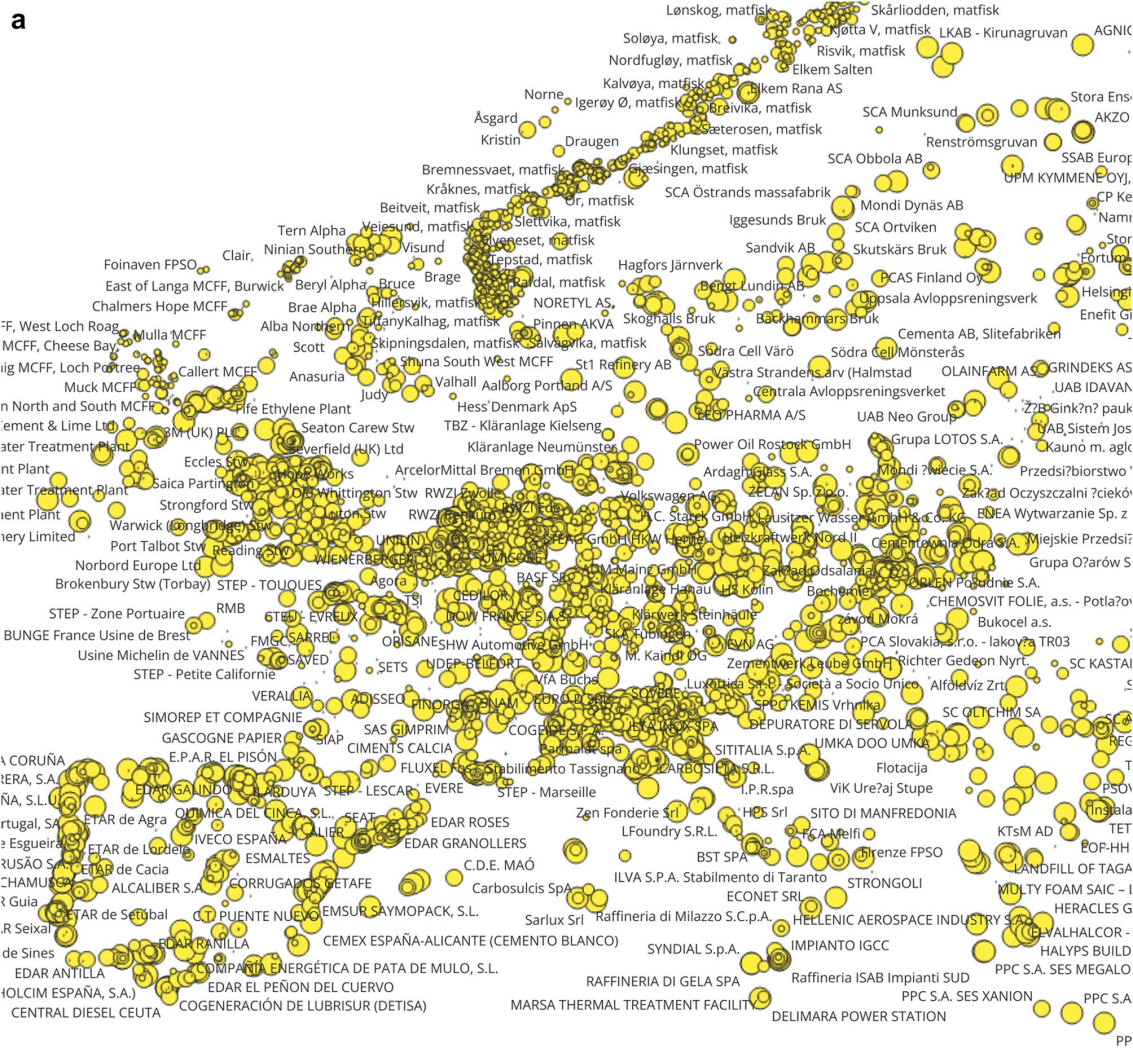

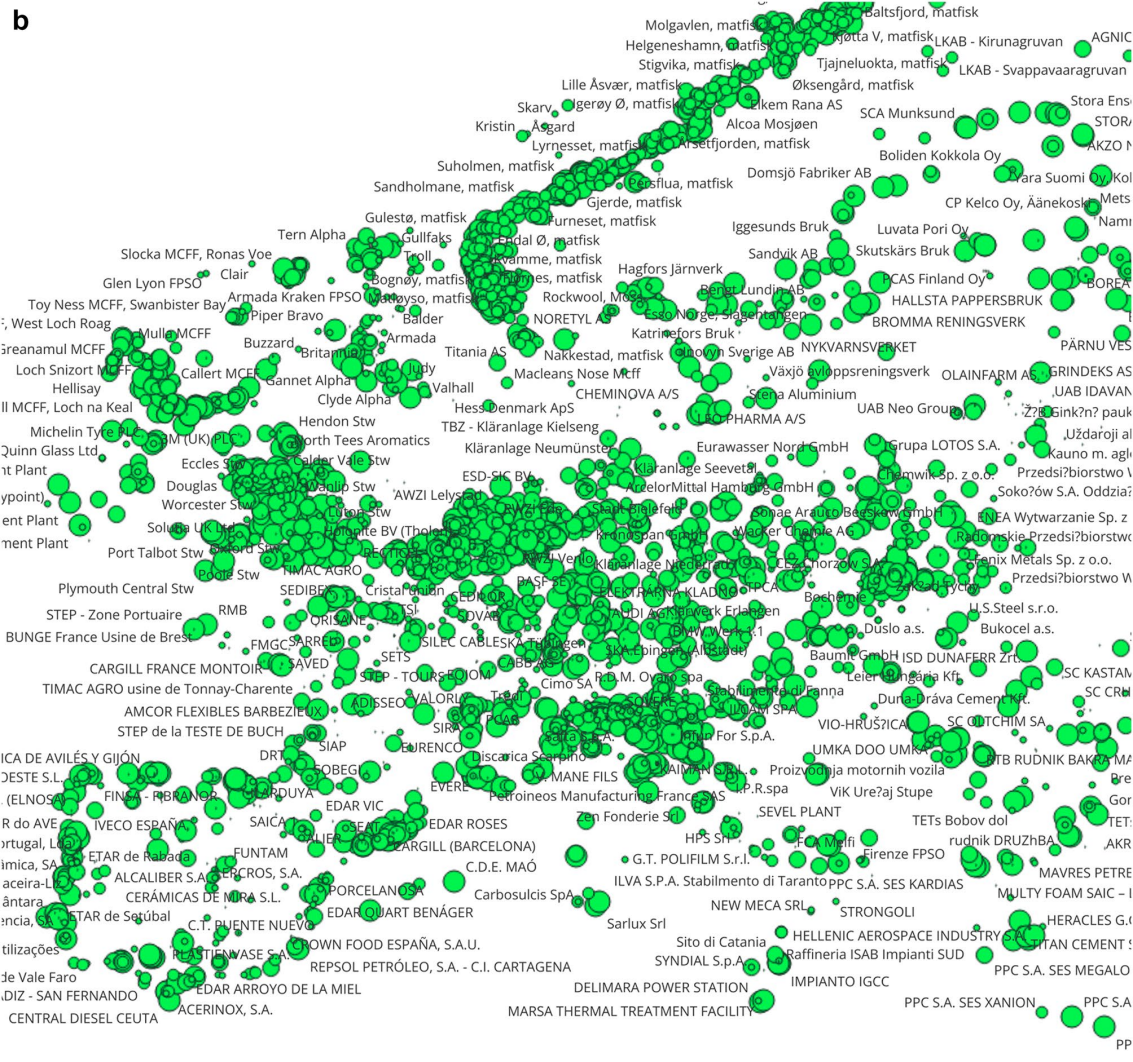

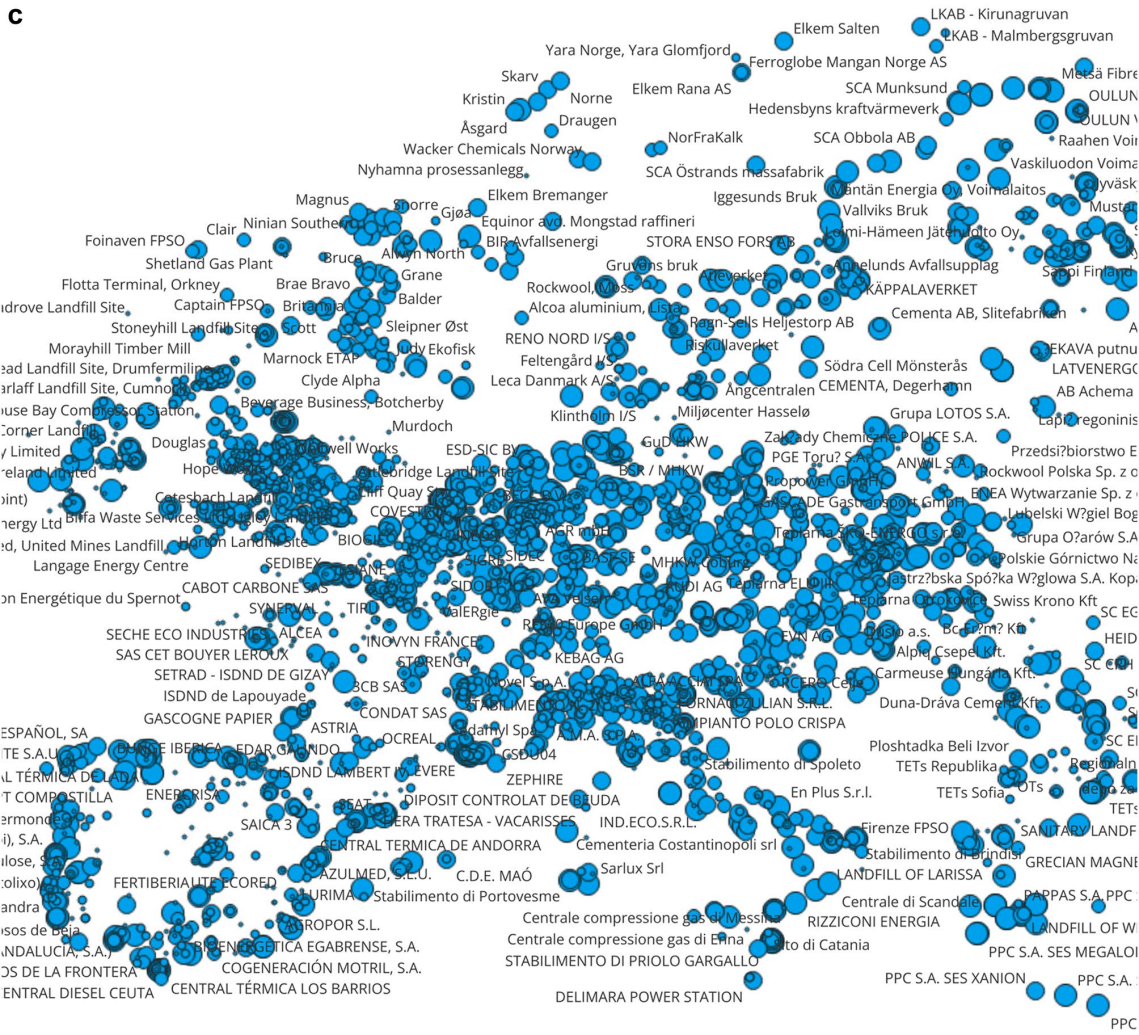
The human toxicity (CTUh, panel **a**), ecotoxicity (CTUe, panel **b**) and global warming ((CO2eq, panel **c**) impact potentials of substances emitted from largest European point sources to air and water in 2017 characterized with USEtox 2.12 and IPCC values. Observations are visualized with QGIS 3.28, Firenze, (https://qgis.org, 2022 version), and point-source releases are sized as a function of the toxicity and global warming impact potentials. *Notes* The outlier data point for Chromium compounds reported by Delimara Power station is corrected as suggested by the European Environmental Agency (693 kg instead of 69,300 kg).

The E-PRTR database contains important information for regional assessment, as companies shall report the geographic coordinates and addresses of facilities together with their country codes. By combining this information with the pollutant release data, we can draw maps of facilities with the largest human toxicity, ecotoxcity and global warming impact potentials (Fig. 2). There are clusters of global warming and toxicity impact potentials in the industrialized regions of Northern England, Northern Italy, the German Ruhr-area, Southern Poland, in the Benelux states, and in coastal areas of Spain, Portugal and the Nordic countries. There is some overlap of areas with the largest toxicity and global warming footprints. In a later section of this study we present findings on the correlation of the different types of impact potentials.

### Facility rankings

An important innovation of our research is that we drill down to the company facility level to investigate which facilities have the largest global warming and toxicity impact potentials in Europe. This way we target stakeholders in sustainable finance and in sustainability in general. Investors, public policy experts and consumers, all need metrics and quantitative information so as to measure sustainability and make informed investment, regulatory and consumption decisions. Current environmental finance data providers (Refinitiv, MSCI, etc.) base their company level sustainability information on reports of listed companies which mainly focus on energy and greenhouse gas emission data and lack the disaggregated geographic, facility level information on human toxicity and ecotoxicity risks.

The facility with the largest contribution to human toxicity and to global warming impact potential is PGE Górnictwo Bełchatów (Table 4). PGE Górnictwo Bełchatów is part of Poland’s largest energy sector company with respect to sales and revenues. The Bełchatów Power Station is a coal-fired power station near Bełchatów, It is one of the largest coal-fired power plants in the world. Hence, it is also among the largest emitters of greenhouse gases. Eight of the top ten polluter facilities in terms of human toxicity are in the Electricity production sector. Most of the largest emitters release mercury compounds in the air.

Several top-ranked facilities in our toxicity assessment are ranked on the top of the 2016 ranking of industrial air pollution by the European Environmental Agency (PGE Górnictwo Bełchatów Nr 1, RWE Power AG Nr 2, LEAG; Kraftwerk Jänschwalde Nr 4, Kraftwerk Boxberg Nr 5, etc.)^22^, which confirms the robustness of our approach. The EEA applied a value-of-life-year (VOLY) estimation technique and calculated marginal damage costs of air pollution. Marginal damage costs for impacts on health have been calculated for heavy metals and organic pollutants.

Presumably it is not surprising that seven of the top ten ranked facilities with the largest contribution to ecotoxicity are in the sewerage and water collection and treatment sector (Table 3). However, he emissions of waste water companies stem from other sectors as well. Furthermore, facilities in the sector of manufacturing other inorganic basic chemicals (Solvay Chimica IT S.P.A.) and in the metal mining sector (Zakłady Górniczo-Hutnicze) released pollutants with the biggest ecotoxicity footprint. Largest emitters in the sewerage sector release mainly zinc compounds, cadmium compounds and nickel compounds into water.

**Table 4:** The facilities with the largest contribution to human toxicity (CTUh), ecotoxicity (CTUe) and global warming potential (CO2eq Kt), emitted from E-PRTR point sources to air and water in 2017, characterized with USEtox 2.12 and IPCC global warming potential values. Only the 10 facilities with the largest impact potentials are shown. Assumptions made in toxicity characterisation are given in the Methods section, and follow Sörme et al. (2016). Calculations were based on the sub-compartment level for toxicity.

No.	Facility	NACE	Country	Human toxicity
1	PGE Górnictwo Bełchatów	Production of electricity	PL	2.91E+03
2	RWE Power AG	Production of electricity	DE	1.38E+03
3	Enefit Energiatootmine	Production of electricity	EE	1.24E+03
4	U.S.Steel s.r.o.	Manuf. iron, steel (...)	SK	1.12E+03
5	TAMEH Polska Sp	Steam, air cond. supply	PL	1.11E+03
6	TETs Maritsa	Production of electricity	BG	9.57E+02
7	LEAG, Kraftwerk	Production of electricity	DE	9.37E+02
8	Zespól Elektrowni	Production of electricity	PL	8.04E+02
9	LEAG Lausitz	Production of electricity	DE	7.78E+02
10	Kraftwerk Boxberg	Production of electricity	DE	7.30E+02

No.	Facility name	NACE	Country	Ecotoxicity
1	Central. postr. za pre. otp. voda u Cve.	Water coll., treat. &supp.	RS	2.63E+10
2	Zakłady Górniczo-Hutnicze	Mining, o. non-ferr. metal ores	PL	2.15E+10
3	Ogranak Termoelektrane A	Trade of electricity	RS	8.26E+09
4	Ebswien hauptkläranlage GmbH	Sewerage	AT	6.59E+09
5	SOLVAY CHIMICA ITALIA S.P.A.	Manuf., other inorg. bas. chem.	IT	4.91E+09
6	Sofiyska prech. stan. za otp.vodi Kubr.	Sewerage	BG	4.81E+09
7	ACQUE VERONESI S.C.AR.L.	Sewerage	IT	4.74E+09
8	EYDAP S.A.	Sewerage	EL	4.36E+09
9	Thames Water Utilities Ltd, Beckton Stw	Sewerage	UK	3.97E+09
10	STATIA EPURARE APE UZATE	Sewerage	RO	3.96E+09

No.	Facility Name	NACE	Country	GHG
1	PGE Górnictwo Bełchatów	Production of electricity	PL	3.76E+04
2	RWE Power AG	Production of electricity	DE	2.99E+04
3	RWE Power AG Kraftwerk Niederaußem	Production of electricity	DE	2.72E+04
4	LEAG, Kraftwerk Jänschwalde	Production of electricity	DE	2.41E+04
5	Kraftwerk Boxberg	Production of electricity	DE	1.92E+04
6	Drax Power Station	Production of electricity	UK	1.81E+04
7	ARCELORMITTAL ATL. et LORR. DNKRQ	Manuf. iron, steel (...)	FR	1.26E+04
8	ENEA Wytwarzanie Sp. z o.o.	Production of electricity	PL	1.21E+04
9	LEAG, Kraftwerk Schwarze Pumpe	Production of electricity	DE	1.15E+04
10	LEAG, Kraftwerk Lippendorf	Production of electricity	DE	1.14E+04

This table shows the aggregated toxicity and global warming impact potentials by the most polluting facilities. The emissions in kilograms ($E_{ijk}$) of substances (i) in the EPRTR have been multiplied by their USEtox 2.12 toxicity characterisation factors ($CF_{ijk}$), by the global warming potential value ($GWP_{i}$) and aggregated across all substances, release media (j) and release sub-media (k). GWP values by greenhouse-gases reported under EPRTR are Methane (CH4): 25 co2eq, Carbon dioxide (CO2): 1co2eq, Hydro-fluorocarbons (HFCs): 7462 co2eq, Nitrous oxide (N2O): 298 co2eq, Perfluorocarbons (PFCs): 9795 co2eq, Sulphur hexafluoride (SF6): 22,800 co2eq. The RWE Power AG human toxicity value in the table is the total reported under two facility names ‘RWE Power AG’ and ‘RWE Power AG Kraftwerk Niederaußem’, and values for Zespól Elektrowni reported under name ‘Zespól Elektrowni P?tnów-Adamów -Konin S.A., Elektrownia Adamów’ and ‘Zespól Elektrowni P?tnów-Adamów -Konin S.A., Elektrownia P?tnów’.

###  Correlation analysis—Global warming potential versus toxicity potential

In LifeCycle Impact Assessment (LCA) the pollutant releases cannot be directly compared after the characterization step, as impact potentials for global warming (CO2eq) and toxicity (CTUe or CTUh) are expressed in different metrics^2^. Thus, a normalization step is needed to convert values into a directly comparable common scale, based on the relative value of the observations to the minima and maxima in the distribution of the characterized values for each year and industry (NACE, Nomenclature of Economic Activities).

Table 5 presents the pairwise correlation ratios of the normalized indicators in our research. Here, we used the lowest, facility level observations for the correlation calculations, but the toxicity and global warming impact potentials were aggregated onto the facility level from the pollutant level. Pairwise correlation ratios of human toxicity, ecotoxicity and global warming potentials are positive in the range of 0.25–0.42, although not very strong.

In general, an important policy lesson from the weak correlation across different impact potentials is that the global warming potential is an important measure of climate change risks, but not a sufficient or complete measure of the overall environmental performance of industrial activities and organizations. We split the sample and calculated correlations for the facilities in the industrial sectors which were identified with the largest toxicity and global warming impacts (electricity production and sewerage) in the subsection of the results on industrial analysis. The correlation ratios in the ’Production of electricity’ sector confirm our earlier results that the association between global warming impact potentials and human toxicity impact potentials is positive and moderately strong (0.57), and therefore in this this sector GHG emission is a better proxy for chemical footprint than in the overall economy. The correlation between the global warming impact potentials and ecotoxicity is, however, weak and negative in the ’Sewerage sector’ giving a further example for the limits of using CO2 data for the overall assessment of environmental performance.

**Table 5:** Cross-correlation (Facility level observations by all sectors and sectors with the largest impact potentials)

Variables	Comparative Toxic Unit (h, n, w)	Comparative Toxic Unit (e, n, w)	CO2eq (n, w)
**All sectors**			
Comparative Toxic Unit (h, n, w)	1.000		
Comparative Toxic Unit (e, n, w)	0.246	1.000	
CO2eq (n, w)	0.422	0.342	1.000
**Production of electricity**			
Comparative Toxic Unit (h, n, w)	1.000		
Comparative Toxic Unit (e, n, w)	0.215	1.000	
CO2eq (n, w)	0.569	0.356	1.000
**Sewerage**			
Comparative Toxic Unit (h, n, w)	1.000		
Comparative Toxic Unit (e, n, w)	0.310	1.000	
CO2eq (n, w)	0.378	− 0.005	1.000

h: comparative toxic unit for human toxicity, e: comparative toxic unit for ecotoxicity, n: normalized values, winsorized values.

### Trends in GHG and toxic emissions

**Figure 3:**
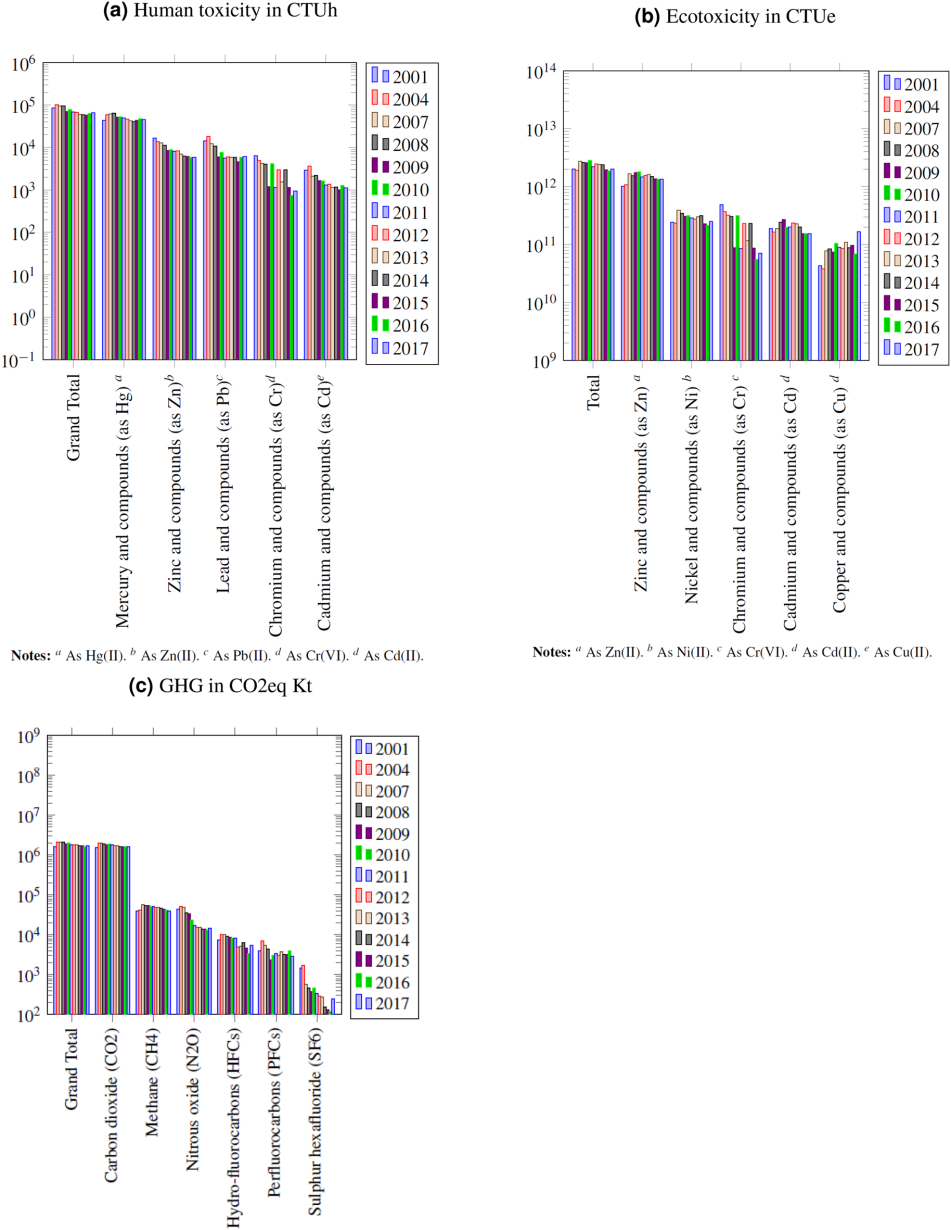
Trend of contribution of substances to human toxicity (CTUh), ecotoxicity (CTUe) and global warming (CO2) impact potentials emitted from European point sources to air and water (2001–2017), characterized with USEtox 2.12. Only the total contribution and the five substances with largest contributions are shown. Assumptions made in characterization are given in the table footnotes. Under the European Pollutant Emission Register (EPER), data was reported every three year, first in 2001 and later in 2004. After 2007 data has been reported every year.

We conducted time-series analysis of the substances with the largest contribution to toxicity as suggested by an earlier paper on Sweden^9^. In the sample period from 2001 to 2017, the first reporting year under the European Pollutant Release and Transfer Register (E-PRTR) was 2007. The E-PRTR followed the European Pollutant Emission Register (EPER) under which reporting was required every three years, first in 2001 and later in 2004. The EPER data is part of the E-PRTR dataset published by the European Environmental Agency, and hence covered in our trend analysis. The slope of the trend of total human toxicity is negative in the early 2000s (Fig. 3a), although became again positive in the last years of the sample in 2016 and 2017. Total human toxicity in the sample decreased by 28%. Human toxicity of zinc was reduced monotonously and radically, while human toxicity of mercury compounds was unchanged.

Although our study focuses on human and freshwater ecotoxicity, mercury can have a wide range of negative health effects on many types of terrestrial animals. Toxic effects include reduced fertility, impaired development of embryos, changes in behaviour and negative effects on blood chemistry^19^.

Earlier research with USEtox 1.01 found that zinc and copper were the substances with the largest contribution to ecotoxicity impact potential in Europe in 2004 (70%, and 30% respectively)^3^. Our calculations confirm the importance of zinc and copper compounds, and shows the increasing and threefold ecotoxicity impact potential for copper. Contrary to the downward trend of human toxicity, ecotoxicity remained unchanged from 2001 to 2017 (Fig. 3b). Another research on the Canadian National Pollutant Release Inventory (NPRI) calculated ecotoxicity potential with the USEtox model in Nova Scotia and showed that copper (51.06%) accounted for the largest share of ecotoxicity potential^8^.

Point source greenhouse gas emissions of industrial facilities increased by 5% from 2001 to 2017. Carbon dioxide releases increased by 7%, while Nitrous oxide releases dropped by 65%, Hydro-fluorocarbons releases by 23% and Sulphur hexafluoride by 83%.

Time trends could be used as a measure of data quality, as substantial dispersion across years could indicate errors in data if assuming no significant change in production or technology^4^. The slopes of the curves were more-or-less stable suggesting stability of data quality except for Chromium. Here, we mention again the reporting error of Chromium releases by the Delimara Power station, highlighted by the European Environmental Agency during the preliminary discussions of the draft paper on Research Square.

### Measurement quality

In this subsection, we investigate the quality of the information in the E-PRTR reports. First we deal with the measurement methods. Second, we analyze the standards used for the reported measurements and calculations.

Facilities are required to report for each pollutant and compartment pair whether releases were measured (“M”), calculated (“C”) or estimated (“E”). “M” is reported when the releases of a facility are derived from direct monitoring results, based on actual measurements of pollutant concentrations for a given release route. “C” is reported when the releases are based on calculations using activity data (fuel used, production rate, etc.) and emission factors or mass balances. “E” is reported when the releases are determined by best assumptions or expert guesses that are not based on publicly available references, or in case of absence of recognised emission estimation methodologies or good practice guidelines. It follows that in general, “M” is considered superior to “C” and “C” superior to “E”.

In the EU-28, about three quarters of the toxic releases are measured, no matter whether impact potentials are calculated as human toxicity impacts or ecotoxicity impacts (Table 6). There is, however, great variation within countries. In Ireland, Italy and Malta a higher share of toxic pollutant releases are estimated by best assumptions or expert guesses that are not based on publicly available references, recognised emission estimation methodologies or good practice guidelines (in terms of ecotoxicity 94%, 29% and 63%, respectively). Four fifth of greenhouse gas emissions are calculated on the basis of fuel used, production rate, etc.

As a consequence, measurement quality may have an effect on data quality and on decisions based on the EPRTR data.

**Table 6:** Shares of measured, calculated and estimated pollutant releases in% of total impact potentials by country (2017)

	AT	BE	BG	CY	CZ	DE	DK	EE	EL	ES	FI	FR	HR	HU	IE	IT	LT	LU	LV	MT	NL	PL	PT	RO	SE	SI	SK	UK	EU 28
	**Human toxicity impact in CTUh in% of the total**																												
Measured$^{\text {a}}$	97	83	100	1	93	73	66	0	61	66	100	91	81	70	72	47	43	100	100	88	62	91	38	9	74	100	99	35	73
Calculated$^{\text {b}}$	3	15	0	99	7	26	34	100	38	30		9	19	30	21	8	57	0		2	38	8	61	90	23	0	1	61	24
Estimated$^{\text {c}}$	0	2	0		1	1	0		1	4		0		0	6	45				10	0	0	1	0	2	0		4	3
	**Ecotoxicity impact in CTUe in% of the total**																												
Measured$^{\text {a}}$	95	86	96	27	97	73	80	15	69	79	100	85	92	30	6	51	97	100	100	24	78	98	83	96	95	99	92	29	69
Calculated$^{\text {b}}$	1	14	4	73	2	25	20	85	18	12		11	8	70	0	21	3	0		14	22	2	5	3	5	0	8	64	22
Estimated$^{\text {c}}$	3	0	0		1	3	0		13	9		4		0	94	29				63	0	0	12	1	0	1		8	9
	**Global warming impact in CO2eq in% of the total**																												
Measured$^{\text {a}}$	16	28	36		54	2	37	2	4	0	100	12	2	57	31	13			100		9	10	3	1	15	4		21	14
Calculated$^{\text {b}}$	82	70	64	100	46	97	62	98	96	98		79	98	42	68	81	100	100		100	81	90	92	96	85	95	100	75	83
Estimated$^{\text {c}}$	1	1	1		0	1	1		0	2		8		1	1	6					10	1	5	3	1	0		3	3

$^{\text {a}}$ **‘Measured’**: “M” is reported when the releases of a facility are derived from direct monitoring results for specific processes at the facility, based on actual continuous or discontinuous measurements of pollutant concentrations for a given release route.

$^{\text {b}}$ **‘Calculated’**: “C” is reported when the releases are based on calculations using activity data (fuel used, production rate, etc.) and emission factors or mass balances.

$^{\text {c}}$ **‘Estimated’**: “E” is reported when the releases are determined by best assumptions or expert guesses that are not based on publicly available references or in case of absence of recognised emission estimation methodologies or good practice guidelines.

Operators should prepare their data collection in accordance with internationally approved methodologies, where such methodologies are available^23^. CEN (Comité Européen de Normalisation, European Committee for Standardization) and ISO (International Organization for Standardization) standards are considered as internationally approved measurement methodologies for toxic releases, For greenhouse gases the “Guidelines for the monitoring and reporting of greenhouse gas emissions under the Emission Trading Scheme”, the “IPCC—Intergovernmental Panel on Climate Change Guidelines” and the “UN-ECE/EMEP Atmospheric Emission Inventory Guidebook” can be reported as calculation methodologies. The operator may only use “equivalent” methodologies other than internationally approved methodologies, if they fulfil one or more of certain criteria: (i) the methodology is already prescribed by the competent authority in a licence / permit for the facility (PER), (ii) A national or regional binding measurement, calculation or estimation methodology is prescribed by legal act for the pollutant and facility concerned (NRB), (iii) the operator has shown that the alternative measurement methodology used is equivalent to existing CEN/ISO measurement standards (ALT), (iv) the operator uses an equivalent methodology and demonstrated its performance equivalence by means of Certified Reference Materials (CRMs), (v) the methodology is a mass balance method (e.g. the calculation of NMVOC releases into air as difference from process input data and incorporation into product) and is accepted by the competent authority MAB), (vi) the methodology is a European-wide sector specific calculation method, developed by industry experts, which has been delivered to the European Commission, to the European Environment Agency, and the relevant international organisations.

Table 7 presents the share of pollutant released by standard types in terms of human toxicity, ecotoxicity and CO2 equivalents. About one third of toxic releases are reported according to the most robust CEN/ISO standards and about one fifth according to the least preferred other methods. Half of the greenhouse gas releases are calculated according to the ETS norms.

**Table 7:** Shares of pollutant released by measurement/calculation standard types in% of the total (in 2017).

	AT	BE	BG	CY	CZ	DE	DK	EE	EL	ES	FI	FR	HR	HU	IE	IT	LT	LU	LV	MT	NL	PL	PT	RO	SE	SI	SK	UK	EU 28
**Human toxicity impact in % of the total**																													
ALT$^{\text {a}}$	0	1	0			0			0	6		7		14	0	2					1	1	3	0	3		0	5	2
CEN/ISO$^{\text {b}}$		13	25	1	29	49		0	60			1	12	0	78	26		100	100	3	22	50	9	7	51	100	99	11	35
CRM$^{\text {c}}$	0	23	0		0	1	0		0	8		7		0		0					1	1	3	0	0	0	0	1	2
MAB$^{\text {d}}$	0	2	0		0	2	0		0	0		1	0	0	21	0		0			0	0	0	0	0	0		23	2
NRB$^{\text {e}}$	13	5	0		58	8	0	100	0	2		16		11	0	11			0		0	7	0	0	0	0	1	0	12
OTH$^{\text {f}}$	54	48	0	0	7	27	0	0	1	43	6	53	70	75	0	46	7	0		97	23	9	33	0	45	0	0	35	21
PER$^{\text {g}}$	33	7	74		6	12	100	0		25	94	14		0	0	15	93				54	31	1	2	1	0	0	23	21
SSC$^{\text {h}}$	0		0		0	0			38	16		1			0	0					0	0	51					3	4
UNECE/EMEP$^{\text {i}}$			0	99	0	0			0				19	0		0		0				0	0	90					1
**Ecotoxicity impact in% of the total**																													
ALT$^{\text {a}}$	0	28	2			7			2	34		6		2	0	8					7	0	11	3	21		0	3	7
CEN/ISO$^{\text {b}}$		9	63	27	79	48		15	67			6	91	0	96	5		100	100	30	5	86	3	51	37	99	94	2	28
CRM$^{\text {c}}$	1	2	2		6	2	0		0	8		3		0		6					12	4	2	5	0	0	0	1	3
MAB$^{\text {d}}$	0	4	0		0	0	0		0	0		1	0	0	0	1		0			0	0	0	0	1	0		1	1
NRB$^{\text {e}}$	75	29	6		13	6	0	83	0	2		2		81	0	24			0		0	4	17	3	0	0	6	0	10
OTH$^{\text {f}}$	14	23	7	0	1	25	0	0	13	33	94	66	1	12	4	40	0	0		70	58	3	57	0	37	1	0	85	39
PER$^{\text {g}}$	10	6	21		0	12	100	1		18	6	13		5	0	16	100				18	2	8	36	4	0	0	8	11
SSC$^{\text {h}}$	0		0		0	0			19	5		3			0	0					0	0	4					0	1
UNECE/EMEP$^{\text {i}}$			0	73	0	0			0				8	0		0		0				0	0	3					0
** GHG impact in% of the total in Co2 equvalent**																													
ALT$^{\text {a}}$		0				0						1		7	3	0					0	0			0			0	0
CEN/ISO$^{\text {b}}$		4	1		0	1			0				0	0	1	0			99		2	0		1	3		14	9	2
CRM$^{\text {c}}$		4			3	0						3		3		0					0	0	0		1	4		2	1
MAB$^{\text {d}}$	4	6			0	1	0		0	14		13	2	5	0	1		38			0	0			10	0		4	3
NRB$^{\text {e}}$	31	10			49	3	3	100		4		7			0	2			1		0	2		0	4	5	0	1	6
OTH$^{\text {f}}$	32	20	9	0	1	18	15		1	12		58	67	54	9	9				100	41	10	16	0	30	1		15	18
PER$^{\text {g}}$	33	14	1		0	5	82	0		61	100	7		2	22	15	100				12	3	4	0	13	0		14	15
SSC$^{\text {h}}$			0		1	16			5	8		9			0	1						0	0					1	5
UNECE/EMEP$^{\text {i}}$			3			0								0		0							0	0					0
ETS$^{\text {j}}$		42	85	100	38	51			93					29	64	70		55			44	85	78	97	39	91	69	52	48
IPCC$^{\text {k}}$				0	8	5							31			2		8			1	0	1	2	0		16		2

$^{\text {a}}$ALT Alternative measurement methodology in accordance with existing CEN/ISO standards.

$^{\text {b}}$CEN/ISO Internationally approved measurement standard.

$^{\text {c}}$CRM (Certified Reference Materials) Measurement methodology for the performance of which is demonstrated by means of certified reference materials and accepted by competent authority.

$^{\text {d}}$MAB Mass balance method which is accepted by the competent authority.

$^{\text {e}}$NRB National or regional binding measurement/calculation methodology prescribed by legal act for the pollutant and facility concerned.

$^{\text {f}}$OTH Other measurement/calculation methodology.

$^{\text {g}}$PER Measurement/Calculation Methodology already prescribed by the competent authority in a licence or an operating permit for that facility.

$^{\text {h}}$SSC European-wide Sector Specific Calculation method.

$^{\text {i}}$UNECE/EMEP UNECE/EMEP EMEP/CORINAIR Emission Inventory Guidebook.

$^{\text {j}}$ETS Emission Trading Scheme Guidelines for the monitoring and reporting of greenhouse gas emissions.

$^{\text {k}}$IPCC Intergovernmental Panel on Climate Change Reference Manual.

## Discussion

The obstacles to the toxicity impact potentials analysis based on the E-PRTR are numerous^9^. One obstacle of our approach is that the number of substances in the E-PRTR is limited, as less than 100 pollutants are on the E-PRTR reporting list, which is well below the 100,000 chemical products listed in the Swedish System of Environmental and Economic Accounting^5^. The E-PRTR list itself is also being revised currently^10^. Another obstacle to precise calculations is that the E-PRTR database does not register emissions below reporting thresholds. Reliability of data could be adversely affected by self-reporting and inappropriate estimations by facilities, and there may be gaps and inconsistencies in reporting across countries?^24^

During the discussion of the draft version of this study on Research Square, the European Environmental Agency informed us that there were some reporting errors in the EEA E-PRTR database, outliers, etc. which the EEA identified as mistakes, but generally cannot change directly. The EEA contacts EU Member States to correct them, but it is their duty to do so, but they sometimes do not correct their data). The EEA identified a big outlier in chromium emissions to water reported by the Delimara Power plant in 2017 (693,000 kg was reported instead of the probable 693 kg). We corrected this error, but similar errors may endanger the usability of the E-PRTR for environmental assessments. The EEA may consider to support the academic community and increase credibility of the E-PRTR by creating a clean data file for researchers. It would help the researchers’ work, and support the national communities to clearly communicate to the national authorities and to the facilities which report inaccurate data.

The E-PRTR data used in our research covers pollutants, which enter the environment from point sources, for example from smokestacks or from discharge pipes of EU facilities. Nonpoint source pollution is more difficult to monitor and neither covered by the E-PRTR database nor by our study. Nonpoint source pollutants are emitted in broader areas usually in small concentration, but can later concentrate after transmitted by meteorological movements or rain, rivers and wind. Motor vehicles, smaller facilities and in general any kind of smaller scale human activities can have unreported emissions^10^. All these can further increase total pollution and toxicity impacts, and hence our calculations underestimate the real toxicity potential. Diffuse emissions of some pollutants have proved to exceed point source emissions in Sweden^4^.

Our methodology followed established research methodologies^4,9^ and calculated the chemical footprint of facilities by the additive aggregation formula. The additive formula is common in the international practice, however, it has relevant consequences^10^. An undesirable feature of additive aggregations is the implied full compensation in a way that high emission in some pollutants can be compensated for by sufficiently low values in other pollutants. An alternative approach would be to use a non-compensatory formula (for example based on a geometric aggregation function) at the facility, industry or national level. Furthermore, the additive toxicity calculation formula in our analysis does not take into account the large number of possible interactions. Especially, the investigation of toxicity consequences from zinc’s interaction with other pollutants could be a potential research direction^10^. A further limitation of the chemical footprint analysis is that the list of pollutants in the E-PRTR and the USEtox model cannot be fully matched.

Precise calculations of environmental footprint with the USEtox model is limited by its uncertainty. The recommended CFs for organic substances have an estimated uncertainty range of up to 2 and 3 orders of magnitude for ecotoxicity and human toxicity, respectively, primarily related to input data^9^. Previous authors have not considered these uncertainties in the calculations, and neither did we. Since metals were recognized as a priority group of pollutants for both human toxicity and ecotoxicity, we remark that all metal CFs are classified as “indicative” in the USETox model following earlier research^9,25^. The related uncertainties have not been quantified, but are larger than the uncertainties associated with organic substances^26^. Despite of all the limitations of the USEtox model, this model is sufficiently documented and has the largest substance coverage and almost full compliance with the science-based criteria^25^. The USEtox is being currently under revision and its new version is under construction. Hence, the European Commission is to decide if and how to take into account its new version^27^.

The usability of our methodology at the company level for environmental assessment or rating is hindered by the well-known problems of company registers. For example, reporting on company ownership or business activity is not mandatory in the E-PRTR regulation, hence aggregation of chemical footprint is not always possible at the final parent company level. Consequently, requirement of reporting parent company, business activity information could help increase the usability of the E-PRTR for environmental rating.

## Methods

**Figure 4:**
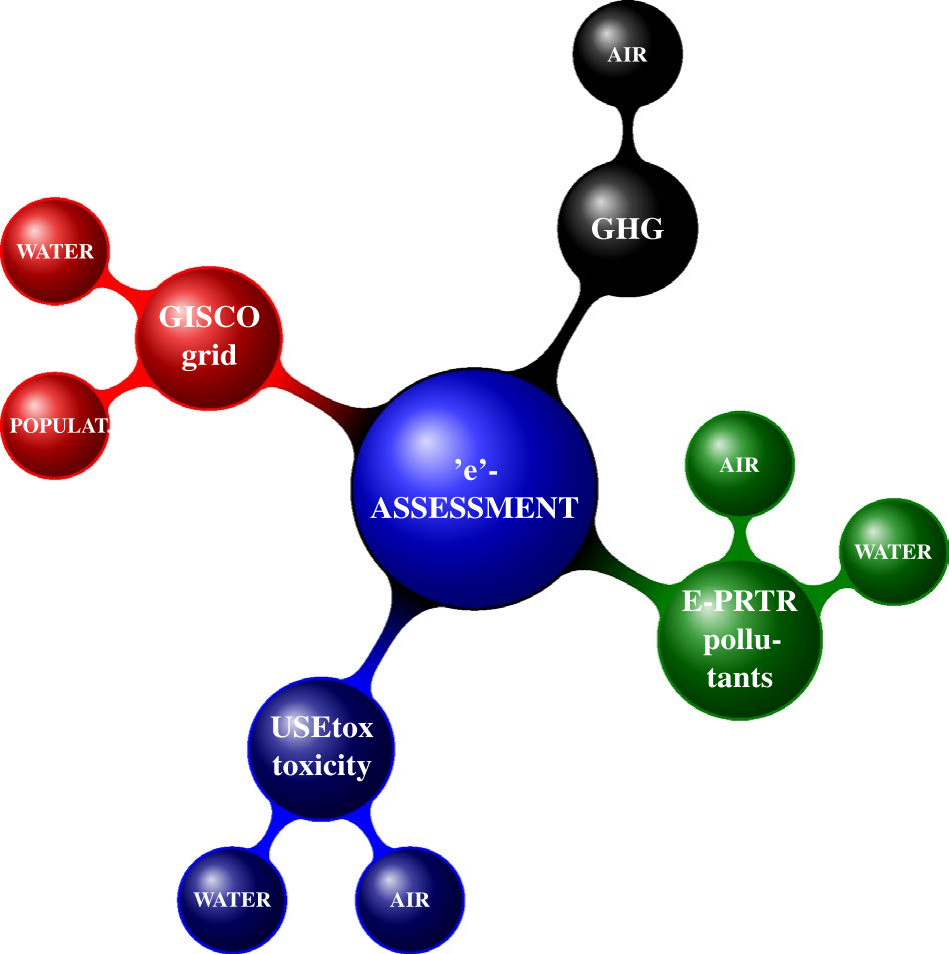
Structure of the environmental assessment.

**Figure 5:**
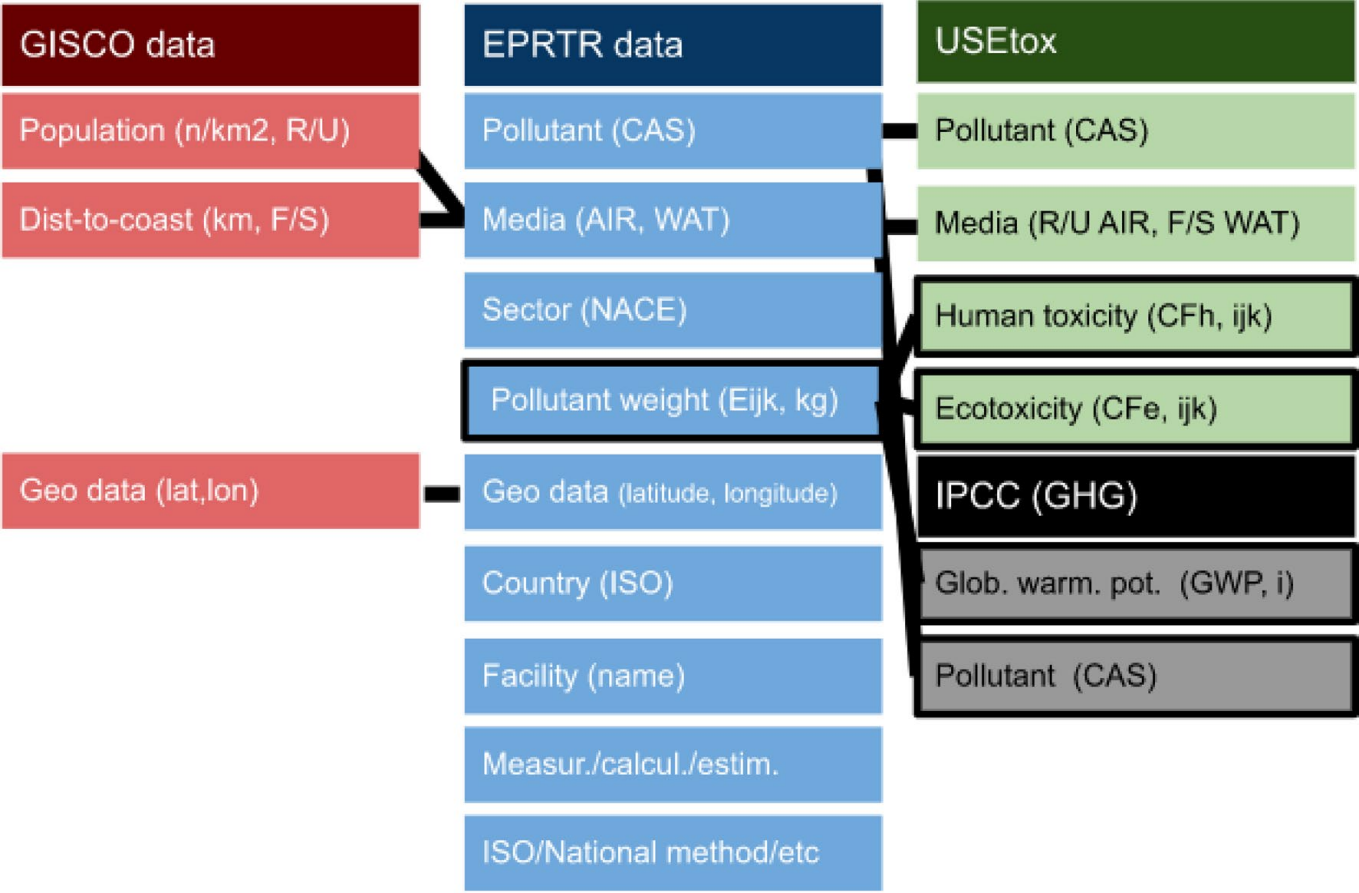
Illustration of connection of the E-PRTR with USEtox model and IPCC GHG global warming potential values. *Notes* CAS—Chemical Abstracts Service registry number, lat—latitude, lon—longitude, F/S—freshwater/seawater, R/U—rural/urban, $CF_{ijk}$—characterization factor for the potential toxicity impacts of the substances released by facility i to media j and submedia k.

We applied the same general method described in earlier research studies in the field^4,9,10^ and calculated toxicity impact potentials for human toxicity and ecotoxicity associated with emissions from point sources in the European Union and in other countries which report to the European Pollutant Register (Figs. 4, 5). The emissions in kilograms ($E_{ijk}$) of substances (i) in the EPRTR have been multiplied by their USEtox 2.12 characterisation factors ($CF_{ijk}$) and aggregated across all substances, release media (j) and release sub-media (k), Eq. (1) as suggested in the USEtox Manual^18^.

1 $$\begin{aligned} Toxicity~ Impact~ Potential= \sum _{ijk}E_{ijk}\times CF_{ijk} \end{aligned}$$

Toxicity impact potentials are measured in Comparative Toxic Units for human health (CTUh) and ecotoxicity (CTUe), respectively. It should be noted that CTUh and CTUe values cannot be directly compared, as they are measured on different scales and in different units.

The characterization factor for human toxicity impacts in this study is expressed at midpoint level (human toxicity potential) in comparative toxic units (CTUh), providing the estimated increase in morbidity in the total human population per unit mass of a contaminant emitted, assuming equal weighting between cancer and non-cancer effects due to a lack of more precise insights into this issue. Its unit is CTUh per kg emitted = [disease cases per kg emitted]. The characterization factor for aquatic ecotoxicity impacts is expressed at midpoint level (ecotoxicity potential) in comparative toxic units (CTUe) and provides an estimate of the potentially affected fraction of species (PAF) integrated over time and volume per unit mass of a chemical emitted. Its unit: CTUe per kg emitted = [PAF $\hbox {m}^{3}$ d per kg emitted].

$CF_{ijk}$ is the characterization factor for the potential toxicity impacts of the substances released by facility i to media j and submedia k (rural air, urban air, freshwater, seawater), and expressed in [cases/kg emitted] for human toxicity impacts and [PAF m3 d/kg emitted] for ecotoxicity impacts at midpoint level and the characterization factor for the potential human health damages [DALY/kg emitted] and for the potential ecosystem quality damages [PDF m3 d/kg emitted].

The quantification of the characterization factors is divided into three calculation steps in the USEtox model^28^. These sequentially provide a fate factor (FF), quantifying how the contaminant is dispersed in the environment, an exposure factor (XF), quantifying human and/or ecological system contact with environmental media, and an effect factor (EF), quantifying effects per kg intake for humans or PAF of aquatic species integrated over the exposed water volume per kg bioavailable chemical in the aquatic environment. The resulting characterization factor that is required for the impact score for either human health or ecological impacts is generally defined as the combination of them, Eq. (2):

2 $$\begin{aligned} CF = FF \times XF \times EF \end{aligned}$$

To further increase the precision of calculations, we draw distinction between different sub-types (k) of compartments (e.g. release media into which the pollutants are released) similarly to an earlier research^10^. Urban air and rural air were differentiated in accordance with the USEtox 2.12 grouping of sub-compartments. In the same vein, freshwater and seawater emissions were also differentiated. Other recent papers on Swedish national chemical footprint^4,9^ assumed that (i) water emissions were freshwater emissions and (ii) air emissions were rural air emissions.

To distinguish point sources emission in urban and in rural areas, we used the GISCO population database from literature^29^. Furthermore, the harmonised European definition of cities and rural areas by literature^30^ was applied, and areas where population density was below 150 inhabitants per $\hbox {km}^2$ were classified as rural, and above 150 inhabitants per $\hbox {km}^2$ as urban. To draw distinction between pollutant releases to seawater and freshwater, we applied a technical rule. We classified emissions into seawater as those pollutant releases into water in the EPRTR database, where the emission point sources’ distance to the seacoast was less than 500 m. All other releases to water for which the distance to seacoast was more than 500 m, were classified as releases into freshwater. The ’Distance to the Nearest Coast’ dataset was also retrieved from the EUROSTAT GISCO database^29^.

Characterization Factors (CFs) were downloaded from the USEtox 2.12 model website (www.usetox.org). Here, both recommended and indicative CFs were used in the calculations, but similarly to previous literature^9,10^ we note that indicative CFs are associated with considerable uncertainties.

USEtox is a generalized model based on scientific consensus providing midpoint characterization factors for human toxicological and freshwater ecotoxicological impacts of chemical emissions in life cycle assessment. USEtox represents best application practice as an interface between ever advancing science and a need for stability, parsimony, transparency, and reliability^18^. Important new features of the latest USEtox model versions include human exposure to pesticide residues in crops; an indoor air compartment for human exposure through inhalation, and improved fate and effect modeling of metals.

Pollutants covered in our research are the same pollutants as in earlier works on Sweden^9,10^. The highest CFs, e.g. the CFs of the most toxic pollutant types were used when the E-PRTR does not provide information on the chemical types of a compound. This assumption is relevant for Cr and As. Also, AOX (Halogenated Organic Compounds) were assumed to be represented by 1,4 di-chlorobenzene, NMVOC by Benzene and PAH (Polyaromatic Hydrocarbons) by Benzo-(a)pyrene. These were chosen as a conservative risk management approach, because they have high CFs and are representative for the group.

Global warming impact potentials of pollutant releases were calculated as recommended by the IPCC. The emissions in kilograms ($E_{i}$) of substances (i) in the EPRTR have been multiplied by their global warming potential value factors ($GWP_{i}$), and aggregated across all substances. Six air pollutants are classified in the EPRTR as GHGs, similarly to the Kyoto Protocol, i.e. Carbon dioxide (CO2), Methane (CH4), Hydro-fluorocarbons (HFCs), Nitrous oxide (N2O), Perfluorocarbons (PFCs), Sulphur hexafluoride (SF6).

3 $$\begin{aligned} Global ~Warming~ Impact ~Potential= \sum _{i}E_{i}\times GWP_{i} \end{aligned}$$

In Life Cycle Impact Assessment (LCA) the “elementary flows” to the environment without further human transformations is translated via a characterization step and aggregated to environmental impact indicator results related to human health, natural environment and resource depletion^2^. With the characterization, impact potential results are expressed in different metrics and hence cannot be compared directly across impact categories. Thus, a “normalization” is performed, converting all impacts to a common unit by calculating the magnitude of the impact indicator results [characterization] relative to some reference information (so-called normalization references)“^31^. Normalized values were calculated with the min-max normalisation^24^. To tackle the problem of time trends and differences across industries the normalized values were calculated in relation to the NACE industry peers in each year for the global warming impact potentials and toxicity impact potentials. Extreme values were treated with the Winsorization technique. We set all outliers at the 95th percentile above the 95th percentile and 5 percentile below the 5th percentile. Such treatment of extreme values is a standard statistical practice before normalisation and recommended by the literature^24^.

## Conclusions

Our study aims at broadening the coverage of company and facility level environmental assessment. Earlier carbon and water footprint methodologies helped assess resource consumption, CO2 emission and related climate change risk separately, but usually neglected integrated assessment of relevant impacts, like those related to the production and use of chemicals^1^.

The seriousness of health, climate or environmental and social consequences cannot be evaluated if only the quantity of pollutants is used^17^. To increase transparency and understanding we characterize the impact of substances by combining their environmental release data with their global warming and toxicity properties (by also controlling for the release media and population density). To implement our identification strategy, we link the European Pollutant Release and Transfer Register (E-PRTR) database to USEtox, a toxicity model based on scientific consensus and to the IPCC global warming impact potential values. USEtox provides midpoint characterization factors for human toxicological and freshwater ecotoxicological impacts of chemical emissions in life cycle assessment.

We assess global warming and toxicity impact potentials in an integrated framework at various aggregation levels from pollutants to industries and company facilities. Furthermore, trend and geographic spread of human toxicity, ecotoxicity and global warming impact potentials are also discussed, which have not been investigated in earlier papers for Europe. Our research aims at supporting the achievement of global environmental policy goals (Sustainable Development Goals, EU Sustainable Finance Taxonomy objectives) by presenting a new set of indicators, which could help operationalize these frameworks on different organizational levels from facilities to companies, industries or nations.

Human toxicity impacts are dominated mostly by mercury compounds in the EU as a whole, accounting for 76% of the total impact potential in 2017. An important risk of further mercury emissions has been already highlighted by earlier research due to mercury’s bio-accumulation properties in organisms and humans during their lifetime. The World Health Organization (WHO) includes mercury in its list of chemicals of major public health concern^20^. Furthermore, zinc compounds and lead compounds are being the pollutants, each with considerable, roughly 10% impact potential of the total on average in the EU.

The pollutant with the largest contribution to ecotoxicity was zinc in 2017 (68% of the total) together with some other metals confirming earlier results^1,3^. The global warming potential impact from industrial point source emissions is mostly related to carbon dioxide releases of the facilities (96% of the total) in EU Member States. Methane emission is the second largest contributor with 4%.

On the industrial level the largest estimated human toxicity footprint was estimated for Production of electricity (46%), followed by Manufacturing of basic iron, steel, and ferro-alloys (20%) and production of cement (10%) in 2017. As for ecotoxicity, sewerage (50%) is the industry with the largest footprint together with Mining of other non-ferrous metal ores (7%).

The large estimated human toxicity and greenhouse gas impact potentials of the electricity production sector further increase the importance of managing the sector’s environmental footprint.

We decomposed the results similarly to an earlier study on Sweden^10^, and found that non-cancer human toxicity dominated the aggregated human toxicity impact potentials in Europe. Still, point source industrial releases of chromium compounds and, Polycyclic aromatic hydrocarbons (PAHs), mercury compounds and PCDD + PCDF (dioxins + furans) have some cancer toxicity impact potential.

By using the geographic coordinates reported in the E-PRTR database by facilities, we present the toxicity clusters in Europe, in particular in Northern England, Northern Italy, the German Ruhr-area, Southern Poland, the Benelux states, and in coastal areas of Spain, Portugal and the Nordic countries can be mentioned. There is some overlap of areas of the largest emissions of greenhouse gases, human toxicity and ecotoxicity.

The facility with the largest contribution to human toxicity in Europe is PGE Górnictwo Bełchatów, a coal-fired power station part of the Poland’s largest energy sector company. Eight of the top ten polluter facilities in terms of human toxicity are in the electricity production sector and release mainly mercury compounds in the air. Ecotoxicity impact potential is also concentrated, six of top ten most toxic facilities is in the sewerage and water collection and treatment sector, and release mainly zinc compounds, cadmium compounds and nickel compounds into water. Several top-ranked facilities in our toxicity assessment are ranked on the top of the 2016 ranking of industrial air pollution by the European Environmental Agency (PGE Górnictwo Bełchatów Nr 1, RWE Power AG Nr 2, LEAG; Kraftwerk Jänschwalde Nr 4, Kraftwerk Boxberg Nr 5, etc. ) confirming the robustness of our approach,

To see whether and to what extent carbon footprint of facilities can be used as an indicator of overall environmental performance, we calculated the pairwise correlation ratios of the normalized indicators in our research. Correlation ratios of human toxicity, ecotoxicity and global warming potentials are positive, although not very strong (in the range of 0.25–0.42). In general, an important policy lesson from the weak correlation across different impact potentials is that the global warming potential is an important measure of climate change risks, but not a sufficient or complete measure of the overall environmental performance of industrial activities and organizations. The correlation between the global warming impact potentials and ecotoxicity is however weak and negative in the ‘Sewerage sector’ giving a further example for the limits of using CO2 data for the overall assessment of environmental performance.

The human toxicity trended downwards in Europe in the early 2000s, although started to increase again in the last years of the sample in 2016 and 2017. Total human toxicity in the sample decreased by 28% from 2001 to 2017. On the pollutant level, toxicity of zinc compounds was reduced monotonously, while human toxicity of mercury compounds was unchanged.

Although the obstacles to the chemical footprint analysis based on the E-PRTR are numerous^9^, we believe that our analysis and methodology help make progress with environmental accounting and transparency.

In this study we also investigate the quality of the information in the E-PRTR reports by using the (i) measurement field in the register and (ii) the field on the standards of used for the reported measurements and calculations. We show that three quarters of the toxic releases are measured, no matter whether impact potentials are calculated as human toxicity impacts or ecotoxicity impacts. There is, however great variation within countries. Also, only one third of toxic releases are reported according to the most robust CEN/ISO standards and about one fifth according to the least preferred other methods.

The preliminary discussion of our study on Research Square revealed that there were some reporting errors in the EEA E-PRTR database, outliers, etc. We argue in this study that the EEA may consider to support the academic community and increase credibility and usability of the E-PRTR by making the currently optional reporting of company information (parent company name, number of employees, hours of operation, etc.) mandatory and by creating a clean data file for researchers and communicate more clearly on the accuracy of the database.

We conclude that the 2.12 USEtox model version produces relatively consistent results with earlier research. Our results are significantly important due to the integrated assessment of global warming and toxicity risks and to the fact that USEtox sub-compartment level toxicity characterisation factors are used for the first time for EU based facilities and matched with point source industrial pollutant releases on the basis of EUROSTAT GISCO population density and distance-to-coast grid data.

Understanding and measuring environmental impacts of individual companies and facilities in an integrated framework for sufficiently large sample of companies is currently needed for environmental accounting, for informed consumer decisions and to scale up financing to clean production and to make progress with the European Green Deal and sustainable finance.

## Supplementary Information

Supplementary Information.

## Data Availability

The study is based on two publicly available data sources: (i) the European Pollutant Release and Transfer Register [E-PRTR database downloaded in March 2020 ], (ii) USEtox 2.12 model [USEtox model download]. Furthermore, the graphs and figures in the study will be publicly available as Supplementary materials and in the Mendeley repository upon publication of the study. Erhart, Szilárd (2023), “Environmental Ranking of European Industrial Facilities by Toxicity and Global Warming Potentials”, Mendeley Data, V1, doi: 10.17632/5drfmgkrz2.1.
